# Combined Modulation of Tumor Metabolism by Metformin and Diclofenac in Glioma

**DOI:** 10.3390/ijms19092586

**Published:** 2018-08-31

**Authors:** Valeria Gerthofer, Marina Kreutz, Kathrin Renner, Birgit Jachnik, Katja Dettmer, Peter Oefner, Markus J. Riemenschneider, Martin Proescholdt, Arabel Vollmann-Zwerenz, Peter Hau, Corinna Seliger

**Affiliations:** 1 Department of Neurology and Wilhelm Sander-NeuroOncology Unit, University Hospital Regensburg, Franz-Josef-Strauss-Allee 11, 93053 Regensburg, Germany; Valeriagerthofer@web.de (V.G.); Birgitt.Jachnik@ukr.de (B.J.); Arabel.Vollmann@ukr.de (A.V.-Z.); Peter.Hau@ukr.de (P.H.); 2 Department of Internal Medicine III, University Hospital Regensburg, Franz-Josef-Strauss-Allee 11, 93053 Regensburg, Germany; Marina.Kreutz@ukr.de (M.K.); Kathrin.Renner-Sattler@ukr.de (K.R.); 3 Institute of Functional Genomics, University of Regensburg, Universitätsstraße 31, 93053 Regensburg, Germany; Katja.Dettmer@ukr.de (K.D.); Peter.Oefner@ukr.de (P.O.); 4 Department of Neuropathology, University Hospital Regensburg, Franz-Josef-Strauss-Allee 11, 93053 Regensburg, Germany; Markus.Riemenschneider@ukr.de (M.J.R.); Martin.Proescholdt@ukr.de (M.P.)

**Keywords:** glioma, BTICs, metformin, diclofenac, lactate

## Abstract

Glioblastoma remains a fatal diagnosis. Previous research has shown that metformin, which is an inhibitor of complex I of the respiratory chain, may inhibit some brain tumor initiating cells (BTICs), albeit at dosages that are too high for clinical use. Here, we explored whether a combined treatment of metformin and diclofenac, which is a non-steroidal anti-inflammatory drug (NSAID) shown to inhibit glycolysis by interfering with lactate efflux, may lead to additive or even synergistic effects on BTICs (BTIC-8, -11, -13 and -18) and tumor cell lines (TCs, U87, and HTZ349). Therefore, we investigated the functional effects, including proliferation and migration, metabolic effects including oxygen consumption and extracellular lactate levels, and effects on the protein level, including signaling pathways. Functional investigation revealed synergistic anti-migratory and anti-proliferative effects of the combined treatment with metformin and diclofenac on BTICs and TCs. Signaling pathways did not sufficiently explain synergistic effects. However, we observed that metformin inhibited cellular oxygen consumption and increased extracellular lactate levels, indicating glycolytic rescue mechanisms. Combined treatment inhibited metformin-induced lactate increase. The combination of metformin and diclofenac may represent a promising new strategy in the treatment of glioblastoma. Combined treatment may reduce the effective doses of the single agents and prevent metabolic rescue mechanisms. Further studies are needed in order to determine possible side effects in humans.

## 1. Introduction

Gliomas are primary brain tumors derived from glial cells or glial precursor cells. The 2016 World Health Organization (WHO) Classification of Tumors of the Central Nervous System differentiates between several entities and four different malignancy grades according to histological and molecular criteria [1]. High-grade gliomas, especially glioblastomas (GBMs, WHO grade IV), rank among the most common and malignant primary brain tumors. GBMs are associated with a poor prognosis. Less than 5% of patients survive five years after being diagnosed; overall survival ranges between 14.6–26.3 months in patients treated within clinical studies [2].

Brain tumor initiating cells (BTICs) represent cancer stem-like progenitor cells, which are characterized by self-renewal, clonogenicity, pluripotency, and the ability to form new tumors after orthotopic implantation [3]. BTICs are not only involved in tumor initiation, they are also involved in progression and recurrence [4,5,6].

Metformin (1,1-dimethylbiguanide hydrochloride, https://www.drugs.com/metformin.html) is the most frequently prescribed drug in the treatment of type-2 diabetes (T2DM) [7]. Besides its anti-diabetic and indirect hypoglycemic effects, it was found that the biguanide drug has antineoplastic effects, and may reduce the risk of several cancer types in diabetic patients [8]. *In vitro* studies disclosed anti-proliferative and anti-migratory effects not only on human glioblastoma (GBM) lines [9], but also glioma-initiating cells [10,11]. Metformin unfolds its action by the inhibition of complex I of the respiratory chain [12]. The adenosin monophosphate/adenosine triphosphate-ratio (AMP/ATP) increases, and AMP-kinase is activated [13,14], whereas the mammalian target of rapamycin (mTOR) is inhibited [10]. In response, rescue mechanisms such as increased glycolysis, and thereby lactate production, are activated [15]. However, most BTICs only respond to high dosages of metformin [16]. The use of metformin in the treatment of T2DM is not significantly associated with a reduced risk of glioma, as recently described by Seliger et al. [17]. Therapeutic effects, including anti-proliferative as well as anti-migratory effects on tumor cells, may underlie different mechanisms of action, and thus have to be distinguished from the questionable protective effects on glioma incidence.

Diclofenac, a non-steroidal anti-inflammatory drug, which is mainly known for its analgesic effects, may inhibit the glycolysis of tumor cells [18]. Epidemiological studies have revealed that the risk of cancer types associated with chronic inflammatory processes may be reduced partly by COX-2 inhibitors [19,20,21,22,23]. In addition to different COX-dependent and independent mechanisms of action, diclofenac is studied as a possible inhibitor of the outward transport of lactate [24]. As a consequence, glucose uptake is reduced, and mitochondrial as well as glycolytic ATP production is inhibited [24,25,26].

The primary aim of our study was to investigate if a combined impairment of mitochondrial respiration and glycolysis by metformin and diclofenac could lead to increased inhibitory effects on BTICs (Figure S1).

## 2. Results

### 2.1. Stem Cell-Like BTICs Express Nestin and SOX 

Using immunocytochemistry, we showed the expression of cancer stem cell markers Nestin and SOX on BTICs. Nestin, which is often expressed in combination with SOX and other stem cell markers, was shown to be expressed on the initiating cells of different tumor types, and was supposed to be a marker for stem cell features such as their self-renewal capacity and tumorigenicity [27,28]. Whereas BTIC-18 was tested positive for Nestin and SOX, BTIC-13 mainly expressed Nestin (Figure S2). 

### 2.2. Combined Treatment of Metformin and Diclofenac Impairs Cell Proliferation and Migration

**Figure 1:**
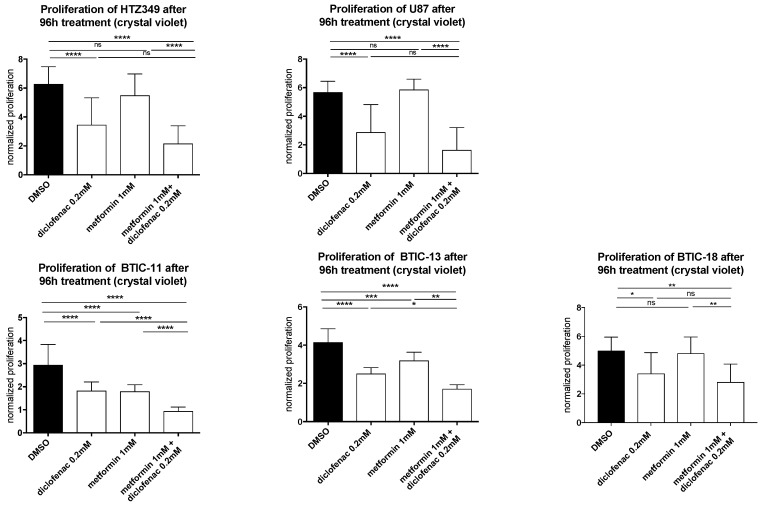
The effects of metformin, diclofenac, and both agents on proliferation were investigated using crystal violet staining at a 96-h time point. Results are expressed as mean ± SD, and were analyzed by two-way ANOVA, *p* = 0.0332 (*), *p* = 0.0021 (**), *p* = 0.0002 (***), *p* ≤ 0.0001 (****) compared pairwise, i.e., the metformin-treated versus metformin and diclofenac conditions.

**Figure 2:**
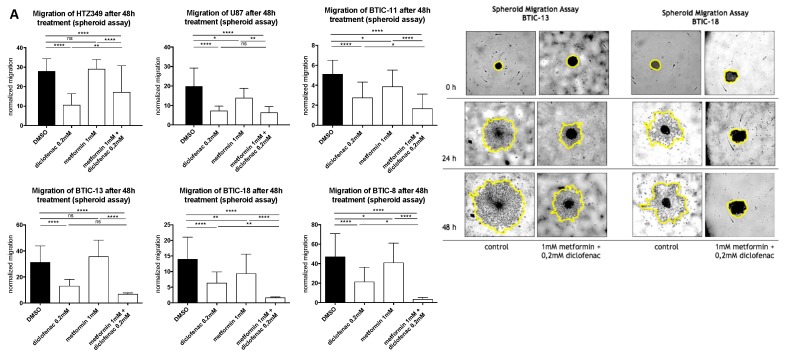
(**A,B**) Spheroid assays were used to analyze the anti-migratory effects at a 48-h time point. Results are expressed as mean ± SD and were analyzed by two-way ANOVA, *p* = 0.0332 (*), *p* = 0.0021 (**), *p* = 0.0002 (***), and *p* ≤ 0.0001 (****) compared pairwise, i.e., the metformin-treated versus metformin and diclofenac condition.

The effects of metformin, diclofenac, and both agents combined with proliferation were investigated using crystal violet staining at 48-h (data not shown) and 96-h time points. Spheroid assays were used to analyze the anti-migratory effects at 24-h (data not shown) and 48-h time points. The early time point was performed to avoid confounding due to excessive proliferation. Metformin was dissolved in medium, whereas diclofenac was dissolved in dimethyl sulfoxide (DMSO), so we performed medium and DMSO controls. Neither control exerted anti-proliferative or anti-migratory effects (Figure S3). After the confirmation of previously described anti-proliferative and anti-migratory effects of high-dose metformin (10 mM, data not shown) and diclofenac (0.2 mM) [11,29], we investigated whether similar effects might be obtained at lower doses by combining both agents. Therefore, we performed proliferation and migration assays applying different doses of metformin (3 × 0.01 mM/day, 0.1 mM, 1 mM) in combination with increasing doses of diclofenac (0.05 mM, 0.1 mM, 0.2 mM, as shown in Figure S4). Compared to DMSO control, the combination of 1 mM of metformin and 0.2 mM of diclofenac significantly reduced cell proliferation in all cells. Comparing the combination to sole metformin treatment, significant effects were observed for both BTICs and TCs. In BTIC-13, BTIC-11, and U87, the anti-proliferative effects of the combined treatment were comparable to high-dosed metformin treatment (10 mM) or treatment that was even more pronounced (Figure S5). Compared to diclofenac treatment, the combined treatment showed a significant reduction of proliferation in BTIC-11 and BTIC-13 (Figure 1). The combination of metformin and diclofenac showed significant anti-migratory effects in all BTIC and TC lines. In BTIC-11, BTIC-18, and BTIC-8, migration was reduced significantly compared to sole metformin as well as to sole diclofenac treatment. In U87 and BTIC-13, the combined treatment led to a slight reduction of migration compared even to high-dosed metformin treatment (Figure 2 and Figure S5), but not compared to diclofenac treatment. Although HTZ349 showed a reduced anti-migratory response upon combined treatment versus 0.2 mM of diclofenac alone in this analysis, the calculation of the combination index (CI) indicated overall synergistic effects. We calculated the CI using CompuSyn software according to the program’s recommendations. Thereby, CI < 1 indicates synergistic effects, CI = 1 additive effects, and CI > 1 indicates antagonistic effects. In the calculation of the combination index, we included all of the tested dosage combinations of diclofenac and metformin. Even though (especially in BTICs) low dosages of metformin and diclofenac didn’t significantly affect proliferation and migration (Figure S4), synergistic effects on proliferation were reached in all of the TC lines (HTZ349 0.83 and U87 0.79) as well as all of the BTICs (BTIC-13 0.95, BTIC-18 0.77, BTIC-11 0.99, and BTIC-8 0.74), according to Ting Chao Chou’s combination index. Regarding migration, synergistic effects were calculated in all of the tested cells except BTIC-13 (HTZ349 0.38, U87 0.56, BTIC-13 >1, BTIC-18 0.68, BTIC-11 0.62, and BTIC-8 0.17, as shown in Table 1).

### 2.3. Metformin, Diclofenac, and Combined Treatment at Low Doses Do Not Increase Cell Death

Measuring cell proliferation using crystal violet staining includes possible confounding by cell attachment and cell death, because several washing steps may lead to the loss of non-adherent cells. Therefore, we performed crystal violet staining on all cell lines with and without laminin coating of the flasks, and added a cell cytotoxicity assay. Laminin is an extracellular matrix glycoprotein and one of the main components of basal lamina that is known to support adhesion and cell proliferation. When seeded with laminin, the treatment response was less efficient, indicating that cell attachment also has an impact on treatment sensitivity, especially in BTICs. As BTIC-8 tends to grow only in spheres, proliferation could only be investigated with laminin coating (Figure S6). However, laminin coating itself may also change pivotal cell characteristics such as progenitor features in BTICs, and the artificial monolayer of cells normally growing fully or partly in spheres may influence cellular programs for proliferation, migration, and adhesion. 

To rule out cytotoxic treatment effects, we afterwards performed a lactate dehydrogenase (LDH) cytotoxicity assay. Compared to medium and the DMSO control, no significant increase in LDH activity was measured after 48 h of treatment (Figure S7). We concluded that metformin, diclofenac, and the drug combination might reduce the cells’ attachment ability without causing cell death. 

### 2.4. Combined Treatment of Metformin and Diclofenac Reduces Extracellular Lactate Levels Compared to Metformin Treatment. Effects on Oxygen Consumption Differ between Cell Lines

**Figure 3:**
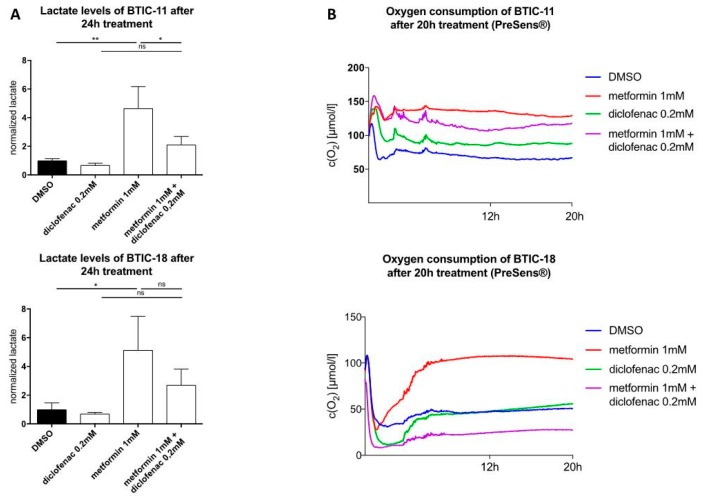
(**A**) Extracellular lactate levels after 24 h of treatment; (**B**) Oxygen concentration in the cell cultures were measured using the SDR SensorDish Reader (PreSens Precision Sensing, Regensburg, Germany) for 20 h. Cells were seeded considering their stereotypic oxygen consumption in different amounts. Measurements were performed in 60-s intervals. Results are expressed as mean ± SD and were analyzed by two-way ANOVA, *p* = 0.0332 (*), *p* = 0.0021 (**), compared pairwise, i.e., the metformin-treated versus metformin and diclofenac condition.

Using the PreSens assay^®^, we were able to confirm that high-dosed metformin (10 mM) decreased oxygen consumption in BTIC and TC cell lines before any obvious alterations in proliferation (Figure S8). Extracellular lactate levels were between 2.5–10 times higher after 48 h of exposure to 10 mM of metformin (Figure S9). Furthermore, extracellular lactate levels were tested at an early time point (24 h). Compared to the control, extracellular lactate levels were four times higher after 24 h of treatment with a low dose of metformin (1 mM). Then, we investigated the changes in extracellular lactate levels and oxygen consumption after exposure to diclofenac and the combination of both drugs. Compared to the DMSO control, diclofenac treatment (24 h) caused a non-significant reduction of extracellular lactate levels. The combination of metformin and diclofenac counteracted the lactate increase in extracellular lactate and concomitant drop in pH that had been observed under the sole treatment with metformin (Figure 3A, Figure S9 exemplarily shown for U87). 

Regarding oxygen consumption, the effects of single and combined treatment were heterogeneous. After 20 h, metformin decreased oxygen consumption in BTICs as well as TCs compared to the DMSO control; dose-dependent effects could be shown. Dependent on the individual BTICs or TCs, diclofenac either decreased (BTIC-11) or increased oxygen consumption (U87, slight time-dependent decrease in BTIC-13), or did not show any alteration (BTIC-18, HTZ349). Combined treatment led to decreased oxygen consumption in HTZ349, U87, BTIC-13, and BTIC-11. After 20 h, oxygen levels were similar to those after the single treatment. An additive inhibitory effect on cell respiration after combined treatment could not be shown. In BTIC-18, a lower oxygen concentration compared to DMSO was measured, indicating an increase in oxygen consumption (Figure 3B, SL8). Those findings suggest the individual cell rescue mechanisms of each cell line after combined treatment.

### 2.5. Combined Treatment Doesn’t Lead to Additive Effects on MTor or STAT3 (Signal Transducer and Activator of Transcription 3) Signaling Pathways

Furthermore, we performed Western blots of the signaling pathways known to be affected by metformin or diclofenac. Seliger et al. and others before were able to show that metformin treatment results in a dose-dependent activation of AMPK, which is an AMP-dependent kinase that is involved in the enzymatic regulation of cellular energy shortages [16]. Subsequently, but also independently, the serine/threonine-kinase mTOR, which is known to be suppressed by AMPK, is inhibited. As previously shown, STAT3 (Signal Transducer and Activator of Transcription 3) is inhibited in a dose-dependent manner after exposure to metformin [30]. Leidgens et al. found that (at least in TCs) STAT3 is also inactivated by diclofenac treatment [29].

**Figure 4:**
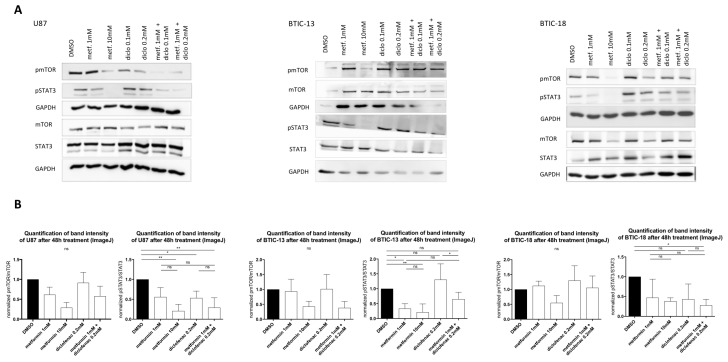
(**A**) Protein levels of the (phosphorylated) mammalian target of rapamycin (p)mTOR, (phosphorylated) signal transducer and activator of transcription 3 (p)STAT3 and GAPDH (glyceraldehyde-3-phosphate dehydrogenase) were investigated using Western blot analysis with specific monoclonal antibodies for STAT3, phosphorylated STAT3 (pSTAT3), mTOR, phosphorylated mTOR (pmTOR) (all Cell Signaling, New England Biolabs GmbH, Frankfurt, Germany), and GAPDH2 (Santa Cruz Biotechnology, Heidelberg, Germany) in dry milk (1%) overnight at 4 °C. Expression was measured by chemoluminescence. (**B**) The intensity of protein bands was determined using ImageJ software, and the protein regulation of at least two replicates was calculated by normalization to loading control and treatment control. Results are expressed as mean ± SD and were analyzed by two-way ANOVA, *p* = 0.0332 (*), *p* = 0.0021 (**), compared pairwise, i.e., the metformin-treated versus metformin and diclofenac condition.

The dose-dependent deactivation of mTOR and STAT3 by metformin could be reproduced in TCs as well as in BTICs (Figure 4). Diclofenac treatment did not show any influences on mTOR activation. Regarding the combined treatment with both agents, pmTOR levels were similar to those of treatment with 1 mM of metformin, whereas in BTIC-13, the decrease of pmTOR was comparable to the effect of high-dosed metformin (10 mM, Figure 4). In U87, diclofenac reduced the activation of STAT3 (phosphorylation), whereas in the other tested cells, pSTAT3 levels did not show any significant alterations compared to the DMSO control. In U87, BTIC-13, and BTIC-18, combined treatment with metformin and diclofenac led to the deactivation of STAT3 without reaching significance. Compared to single treatment with metformin and diclofenac, no additive effects were shown.

## 3. Discussion 

For the first time, we showed that metformin in combination with diclofenac reduced migration and proliferation in primary human brain tumor initiating cells, as well as in human glioma cells. The effects on BTICs were weaker after coating with laminin. Besides a possible cell attachment effect, laminin is also known to modify the stem-cell characteristics of cancer stem cells. For instance, Qin et al. reviewed the role of the extracellular matrix protein as a regulator of cancer stem cell properties such as epithelial–mesenchymal transition, dedifferentiation, and metastatic potential [31]. According to that, we showed that the proliferation of U87 and HTZ349 as differentiated glioma cells was inhibited, regardless of laminin addition. 

As LDH activity was not increased after the treatment, we ruled out cytotoxic effects and the possibility that stronger treatment response without laminin coating might be due to cell death and thereby a loss of adherence. 

We were able to confirm that metformin treatment reduced oxygen consumption and increased extracellular lactate levels, indicating intensified energy generation via glycolysis, whereas diclofenac only slightly reduced lactate. The reduction of extracellular lactate levels by diclofenac was less pronounced in comparison with previous publications exploring the inhibition of cellular lactate production by NSAIDs. This might be because BTICs especially frequently rely on mitochondrial ATP production in an oxygen-rich cell culture environment. Regarding the different BTICs used in this study, particularly untreated BTIC-18 consumed high amounts of oxygen. In accordance, metformin treatment led to a strong inhibition of oxygen consumption, whereas diclofenac did not alter extracellular lactate levels significantly. The combination of both drugs reduced extracellular lactate levels compared to single metformin treatment. Regarding cell-signaling pathways, the combined treatment led to a reduction of activated (phosphorylated) STAT3 in U87, whereas in BTICs, the combination did not alter phosphorylated STAT3 (pSTAT3) levels compared to the single treatment with metformin or diclofenac. 

A large number of epidemiological studies revealed that exposure to metformin was associated with a reduction of the risk of different cancer types such as liver, colorectal, pancreatic, stomach, and esophageal cancer [8]. However, there were also numerous malignancies that did not benefit from metformin exposure. Seliger et al. showed that metformin treatment did not significantly reduce the risk of glioma [17]. Besides epidemiological associations between metformin and the risk of cancer, the drug’s antineoplastic effects have been observed in many in vitro and in vivo studies. Regarding brain tumor initiating cells, metformin inhibited migration [32] and proliferation by inducing apoptosis, autophagy, and differentiation [10,11,13,33,34]. Würth et al. demonstrated that metformin exerts its anti-proliferative effects, especially on CD 133-expressing BTICs, without causing cell death up to 10 mM [10]. In addition, metformin showed anticancer effects in many in vivo experiments. More than 15 years ago, Owen et al. were able to show that metformin inhibited the mitochondrial respiratory chain, and thereby restrained gluconeogenesis in a time-dependent manner [12]. In BTICs, impaired proliferation and migration was also due to an inhibition of complex I of the respiratory chain [9,16]. In order to survive, malignant cells need to activate alternative ways of ATP production, similar to increased glycolysis resulting in dropping pH values in the cell culture medium [33]. The activation of metabolic rescue mechanisms, the differentiation of progenitor cell-like brain tumor initiating cells into non-tumorigenic cells, as well as the suppression of inflammatory response, are exerted through the activation of AMP-dependent kinase (AMPK) [7,10,13,14,35,36] and the downstream inhibition of mTOR [9,11,16]. Furthermore, metformin treatment exerts its effects partly through the deactivation of STAT3 [30]. STAT3 has been shown to be essential to maintain the tumor-initiating capacity, invasion capacity, cell survival, and cell cycle progression [37,38]. Metformin subsequently induces apoptosis and autophagy, and blocks tumor progression [39,40,41]. That the use of metformin in the treatment of T2DM did not show protective effects on glioma incidence might be explained by different underlying molecular mechanisms compared to therapeutic use in cancer treatment.

In daily T2DM treatment, a drug dose of up to 2550 mg metformin/day is recommended by the Federal Drug Administration (FDA) (https://www.accessdata.fda.gov/drugsatfda_docs/ label/2017/020357s037s039,021202s021s023lbl.pdf), and with those doses, drug levels of around 40 µM can be measured in the portal vein and cerebrospinal fluid, and 10 µM can be measured in brain tissue, whereas in mouse models with long-term metformin use and intensified dosages, metformin concentrations of up to 5 mM could be reached in the serum, which extended the lifespan of mice [42,43,44]. Although in most of the prior in vitro studies (among others, Gritti et al.) metformin was used in even higher dosages [34], some authors have argued that metformin may have several effects that are individually small but collectively effective at achieving the desired effect. To reduce the individual drug doses, metformin is currently combined with different pharmacological partners or used in combination with classical chemotherapeutics in order to reduce drug doses and possibly side effects by maintaining anticancer effects. Metformin and temozolomide, which is an alkylating agent used in the treatment of WHO grade IV glioma according to the Stupp protocol [45], synergistically inhibited the proliferation of brain tumor-initiating and differentiated glioma cells in vitro and in vivo. Recently, Valtorta et al. showed that the combined treatment of metformin and temozolomide helped to overcome resistance in glioblastoma multiforme [46]. According to Aldea et al., metformin in combination with sorafenib exerted selective anti-proliferative effects in BTICs and non-stem glioblastoma cells, whereby sorafenib also affected the proliferation of normal cells [47]. Several studies showed that anti-proliferative effects on different types of cancer could be potentiated by combining metformin with classic chemotherapeutic drugs. For example, Zhu et al. treated cholangiocarcinoma cells with metformin and gemcitabine or cisplatin, Peng et al. used the biguanide in combination with gefitinib on bladder cancer cells, and Harada et al. demonstrated that metformin in combination with 5-FU (fluorouracil) suppressed tumor progression in squamous cell carcinoma [48,49,50]. We used a dose of 1 mM of metformin in most of our assays, which can still be considered as high, because complex 1 in hepatocytes was started to be inhibited at doses as low as 50–100 µM of metformin [12,51]. Furthermore, in recent experiments, 1 mM of metformin has been shown to be associated with increased cell apoptosis in HUVEC (human umbilical vein endothelial cells) [52].

In 1956, Otto Warburg revealed that cancer cells provided their energy mainly by upregulating glycolysis despite sufficient oxygen supply in the tumor microenvironment [53]. Today, this metabolic switch from the oxidative to the glycolytic pathway is known as the Warburg effect. As metformin promotes glycolysis and in consequence lactate production as a rescue mechanism, glycolysis-blocking agents seem to be suitable pharmacological partners. Saber et al. showed that metformin in combination with 5-aminosalicylacid decreased the proliferation of colorectal cancer and in melanoma, the combination of metformin and oxamate, known as an inhibitor of LDH and thereby glycolysis, retarded tumor progression in mice [54,55]. 

In this study, we considered the frequently used NSAID diclofenac as a promising combination partner. Besides its anti-inflammatory and analgesic capacities (www.drugs.com/pro/diclofenac.html), diclofenac and other NSAIDs have been shown to exert antineoplastic effects by COX-dependent and independent mechanisms [22,56,57,58]. Among others, Cha and Taketo reviewed the important role of COX-2 and its product PGE2 in tumorigenesis, including angiogenesis, migration, invasion, tumor progression, the inhibition of apoptosis, and in consequence, the inhibitory effects of NSAIDs [20,21,59,60,61]. Chirasani et al. demonstrated in a murine glioma model that diclofenac inhibited lactate formation beginning at a dosage of 0.1 mM [18]. According to Gottfried et al., diclofenac may affect glucose uptake and lactate secretion not only by reducing the gene expression of responsible transporters via MYC, but also by inhibiting lactate efflux [24]. Sasaki et al. demonstrated that out of the four tested NSAIDs, diclofenac was the most potent inhibitor of glycolysis [62]. 

Constant lactate efflux is essential to avoid intracellular acidification and guarantee continuous ATP production via glycolysis relying on a concentration gradient of lactate and protons between the intracellular compartment and the tumor microenvironment [63].

In summary, we provide data that suggest that a combination of increased intracellular lactate levels due to the inhibition of mitochondrial respiration and a blockage of glycolysis exerts additive anti-proliferative and anti-migratory effects of metformin and diclofenac in BTIC and glioma cell lines. These effects are not explained by the direct effects of metformin on candidate signaling pathways. As diclofenac is assumed to reduce extracellular lactate levels not only by the direct inhibition of outward transport, but also by influencing gene expression, further studies are needed to investigate the possible additive effects on those signal pathways. 

As especially anti-migratory effects were shown, it might be interesting to observe how signal pathways that are involved in migration and metastasis formation, i.e., NF-kappaB (Nuclear Factor kappa B) or SMAD, are affected by the combined treatment. Furthermore, in vivo, BTICs can be differed into a proliferative and migrative subtypes [64]. Those different subtypes might react in different ways to the combined treatment used in this study. Human mesenchymal stem cells isolated from the umbilical cord only showed a 20% reduction of viability after being treated with 50 mM of metformin, but we don’t know how healthy cells are affected by the combined treatment [10]. Impairing mitochondrial respiration and glycolysis might affect other cells beyond cancer cells. Control cell lines, i.e. astrocytes, are needed, as a therapy used in cancer treatment is expected to be active on pathological cells while not affecting normal cells. Furthermore, metformin, especially in combination with diclofenac, doesn’t exert its effects on the human body only by impairing a single biochemical pathway. Used in therapeutic dosages, the risk of causing a lactic acid crisis might be more relevant than in diabetic treatment. Besides, the effects of metformin may vary according to short-term or long-term exposure, which we did not further check in our experimental setting [65,66]. In order to investigate not only the exact mechanism of a combination of metformin and diclofenac, but also its effects on the tumor microenvironment, including angiogenesis and the immune system, as well as the whole (human) body, in vivo models are needed. If these models can substantiate the effects we show here, future clinical trials may investigate metformin in combination with diclofenac in patients with glioblastoma. 

## 4. Materials and Methods

### 4.1. Ethics Statement

The local neuropathology department determined the patients’ diagnoses and WHO grade, and routine histopathology was accompanied by testing for *IDH1* mutation (by Sanger or Pyrosequencing) and *MGMT* promoter methylation status (by MethyQESD [67]). Clinical parameters such as age, gender, type of treatment, and overall survival (according to the RANO criteria) were available for all patients. The ethics committee of the University of Regensburg, Regensburg, Germany (No° 11-103-0182) approved the study, and all of the patients gave written informed consent.

### 4.2. Tumor Cell Lines

BTIC-8, -11, -13, and -18 are primary tumor cell cultures derived from the same human glioblastoma, as described before [11,29]. For the enrichment of BTICs, tumor specimens were mechanically or enzymatically dissociated, washed with PBS (Phosphate-buffered saline), and passed through a cell strainer with a 30-µm pore size to obtain a single cell suspension. After isolation, BTICs were frozen and used at different time points in passages 12–25. BTICs were maintained in RHB-A-based serum-free culture media supplemented with 20 ng/mL of the mitogens EGF (Epidermal Growth Factor) and FGF (Fibroblast Growth Factor) (both Miltenyi Biotech, Bergisch Gladbach, Germany), at 37 °C, 5% CO_2_, 95% humidity in a standard tissue culture incubator. The progenitor features of BTIC lines were verified by clonogenicity assays, and partly by tumor take assays in an immunocompromised mouse model. 

Human high-grade glioma cell line U87 was obtained from the American Type Culture Collection (Manassas, VA, USA). HTZ349 is a primary tumor cell culture derived from the resection of a human glioblastoma established at the university clinic in Würzburg, Germany, as described before [68]. Tumor cells were maintained as monolayer cultures in Dulbecco’s Modified Eagles Medium (DMEM with 1 g/L of glucose; Sigma-Aldrich, Taufkirchen, Germany), supplemented with 10% fetal calf serum (FCS; Biochrom, Berlin, Germany) at 37 °C, 5% CO_2_, 95% humidity in a standard tissue culture incubator. All of the experiments were performed using Dulbecco’s Modified Eagles Medium, supplemented with 10% fetal calf serum for TCs, 2% of B27, and 0.01% of EGF and FGF for BTICs.

### 4.3. Proliferation Assay

Proliferation was assessed using crystal violet staining. Briefly, cells were seeded at densities of 2.5 × 10^3^ cells/mL in a 200 µL/well. In addition, proliferation was also assessed with laminin coating. Therefore, non-adherent as well as adherent cells were seeded on laminin-coated wells and incubated for at least 2 h. After 48 h to 72 h, the media was renewed, and cells (at least five replicates) were treated with specific concentrations of metformin, diclofenac, a combination of both, or control media. Proliferation was measured at 0 h, 48 h, and 96 h, respectively. Medium was exchanged with 0.5% of crystal violet in a 20% methanol solution, and cells were stained for 10 min. After washing and drying, the crystal violet was diluted into a homogenous solution by the addition of 0.1 M sodium citrate in 50% ethanol, and measured at 550 nm (VarioSkan Flash Multimode Reader, Thermo Scientific, Waltham, MA, USA). For all of the assays, background fluorescence was subtracted, and values were normalized to the 0 h values. Assays were performed in five or six replicates and repeated twice.

### 4.4. Migration Assay

Tumor spheroids were generated by seeding 2.5 × 10^3^ cells onto agarose-coated wells (1% agarose in 1 × PBS), as described [11,29]. Cells were cultured for 48 h to allow spheroid formation. Mature spheroids were transferred into non-coated 96-well plates containing the corresponding drugs. Cell migration was monitored at 0 h, 24 h, and 48 h, taking into account the earliest time point when migration was measurable to prevent the dilution of results by proliferation effects. The area covered by cells was measured by tracing the covered area manually (freehand selection module, ImageJ software, version 1.50i, NIH, USA). Assays were performed in five to six replicates and repeated twice.

### 4.5. Protein Isolation and Western Blot

To investigate the protein levels of (p)mTOR, (p)STAT3, and GAPDH, whole-cell lysates were prepared with RIPA buffer (radioimmunoprecipitation assay buffer). For Western blot analysis, 30 µg of total cell lysates were diluted in Laemmli buffer, separated on a 10% SDS-PAGE gel, and transferred to nitrocellulose membranes by semi-dry blotting or wet blotting. The membranes were blocked with 5% milk powder or 5% BSA (bovine serum albumin) in 0.02% Tween in TBS (Tris-buffered saline) for 1 h. Membranes were incubated with specific monoclonal antibodies for STAT3 (#9145), phosphorylated STAT3 (#4904) (pSTAT3) (#9145), mTOR (#2983), phosphorylated mTOR (pmTOR) (#5536) (all Cell Signaling, New England Biolabs GmbH, Germany), and GAPDH2 (sc-48167) (Santa Cruz Biotechnology, Heidelberg, Germany) in dry milk (1%) overnight at 4 °C. For U87 and BTIC-18, we used different membranes for phosphorylated and unphosphorylated proteins. For BTIC-13, the same membrane was used for (p)mTOR and (p)STAT3. After incubation with the first antibody (phosphorylated), expression was measured; afterwards, the membranes were stripped and incubated with the secondary antibody (unphosphorylated). Expression was measured by chemoluminescence (ECL Western Blot Bright, Biozym, Germany). Intensities of protein bands were measured with ImageJ software using the gel analyze module, and protein regulation of two or three Western blots each was calculated by normalization to loading and treatment control using GraphPad Prism software (Version 6 and 7, GraphPad Software, La Jolla, CA, USA). 

### 4.6. LDH Activity Measurement

To check for LDH enzyme activity, the LDH cytotoxicity assay (Promega, Mannheim, Germany) was performed. The assay is based on an enzymatic coupling reaction: LDH oxidizes lactate in order to generate NADH, which then reacts with pyruvate and a dye to generate a yellow color. LDH activity was quantified with a plate reader (VarioSkan Flash Multimode Reader, Thermo Scientific, USA) at 490-nm absorption. In summary, cells were seeded 24 h prior to treatment with 2.5 × 10^3^ cells/well in 200 µL of serum-free media/well, and incubated with either the indicated concentrations of metformin, diclofenac, or a combination of both; NaOxamat (25 mM) was used as a positive control. Then, 24 h and 48 h later, LDH activity was measured.

### 4.7. Online Measurement of Oxygen Concentration and PH Levels in Cell Culture

The SDR SensorDish Reader (PreSens Precision Sensing, Regensburg, Germany) is a 24-channel oxygen and pH meter. Therefore, luminescent dyes integrated at the bottom of a 24-well multidish function as sensors. The lifetime of those dyes depends on the oxygen concentration in each well. Depending on the pH levels, the fluorescent dyes change their intensity over time. 

The signals are read out non-invasively by the SensorDish Reader and converted using calibration parameters stored in the software. Cells were seeded considering their stereotypic oxygen consumption in different amounts, i.e., 1.5 (BTIC-18) or 3 × 10^5^ (BTIC-11, BTIC-13, BTIC-8, U87, and HTZ349) cells/well and incubated in 1 mL of medium. After 24 h, medium was exchanged and cells were treated with metformin, diclofenac, or a combination of both agents. Afterwards, the SensorDish Reader was used in the incubator for the whole duration of the 24-h treatment period.

### 4.8. Extracellular Lactate Levels

Extracellular lactate was measured using mass spectrometry, as described [69].

### 4.9. Combination Index

Ting Chao Chou’s combination index was automatically calculated using CompuSyn software (www.combosyn.com). The combination index allows differing between additive, synergistic, or antagonistic effects of a drug combination using the dose-effect curve for each drug and the combined treatment. Therefore, the dose-effect parameters of each drug alone, as well as in combination, are needed in order to determine the CI value.

Four data points of 3 × 0.01 mM, 0.1 mM, 1 mM, and 10 mM, and three data points of 0.05 mM, 0.1 mM, and 0.2 mM were used to determine the dose-effect curve of metformin and diclofenac. Furthermore, 3 × 0.01 + 0.05, 3 × 0.01 + 0.1, 3 × 0.01 + 0.2, 0.1 + 0.05, 0.1 + 0.1, + 0.2, 1 + 0.05, 1 + 0.1, and 1 + 0.2 were used in combination analysis.

Detailed procedures of automated dose-effect analysis for the quantification/simulation of synergism or antagonism are given in the User’s Guide for CompuSyn [70].

### 4.10. Immunocytochemistry

For the staining of DAPI (4′,6-Diamidin-2-phenylindol), Nestin and SOX 2–5 × 10^5^ (U87: 5–10 × 10^3^) cells/well were seeded in laminin-coated coverslips. Supernatants were removed 48 h after treatment, and cells were fixed with 4% paraformaldehyde (441244, Sigma-Aldrich, Taufkirchen, Germany) for 10 min. After threefold washing with PBS and blocking with blocking buffer (PBS, 10% donkey serum #S30-100, Merck-Millipore, Darmstadt, Germany, 1% BSA #82-100-6, Merck-Millipore, Darmstadt, Germany, 0.1% Triton X-100 #T8787, Sigma Aldrich, Taufkirchen, Germany) for 2 h, coverslips were incubated with primary antibodies diluted in blocking buffer (mouse anti-Nestin #MAB5326, Merck-Millipore, Darmstadt, Germany ,1:500; goat anti-sox-2 #sc-17320, Santa Cruz, Dallas, TX, USA, 1:500; mouse anti-MCT-1 #sc-365501, Santa Cruz, Dallas, TX, USA, 1:50; rabbit anti-MCT-4 #sc-50329, Santa Cruz, Dallas, TX, USA, 1:50) at 4 °C overnight, washed with blocking buffer three times before incubation with secondary antibodies (Alexa Fluor^®^ 568 donkey anti mouse #A-10037, Life technologies, Darmstadt, Germany, 1:500; Alexa Fluor^®^ 488 donkey anti-rabbit #A-21206, Life technologies, Darmstadt, Germany, 1:1000; Alexa Fluor^®^ 488 donkey anti-goat #A-11055, Life technologies, Darmstadt, Germany, 1:1000), and DAPI (#D9542, Sigma-Aldrich, 1:1000) for nuclear staining for 1 h. Coverslips were also washed three times with blocking buffer and PBS, respectively, before they were mounted with Prolong Gold (# P96930 , Invitrogen, Carlsbad, CA, USA) and dried overnight. Fluorescence microscopy was performed using the Zeiss Axio Observer.Z1 microscope (Zeiss Axio Observer.Z1, Visitron Systems GmbH, Puchheim, Germany). 

### 4.11. Statistics

Analyses of significant differences between treatment groups (mean values and SDs) were performed by two-way ANOVA with Tukey’s multiple comparisons test with a 95% confidence interval, *p* = 0.0332 (*), *p* = 0.0021 (**), *p* = 0.0002 (***), *p* ≤ 0.0001 (****). After being normalized, every treatment/control was compared to every other treatment/control. Data were analyzed using GraphPad Prism software (version 6 and 7, GraphPad Software, USA).

## Figures and Tables

**Table 1:** Combination index (CI) using CompuSyn software. Thereby, CI < 1 indicates synergistic effects, CI = 1 additive effects, and CI > 1 antagonistic effects.

	CI-Proliferation	CI-Migration	Effect-Proliferation	Effect-Migration
HTZ 349-laminin	0.83699	0.38254	synergistic	synergistic
BTIC-13-laminin	0.95719	>1	synergistic	antagonistic
BTIC-18-laminin	0.77451	0.68924	synergistic	synergistic
U87-aminin	0.7953	0.56451	synergistic	synergistic
BTIC-11-laminin	0.99912	0.62748	synergistic	synergistic
BTIC-8-laminin	0.74232	0.17014	synergistic	synergistic

## Supplementary Materials

The following are available online at http://www.mdpi.com/1422-0067/19/9/2586/s1, Figure S1: Graphical abstract summarizing the expected mechanism of action of combined metformin and diclofenac treatment, Figure S2: Immunocytochemical expression of cancer stem cell-markers Nestin and SOX, Figure S3: (**A**) The effects of medium control and DMSO on proliferation were investigated using crystal violet staining at a 96 h time point. (**B**) Spheroid assays were used to analyze anti-migratory effects at a 48 h time point, Figure S4: Cell proliferation of HTZ349 and BTIC-13 after 96 h treatment with different dosages of metformin, diclofenac and both agents was investigated using crystal violet staining, Figure S5: The effects of metformin, diclofenac and both agents on proliferation were investigated applying crystal violet staining at a 96-h time point, Figure S6: The effects of metformin, diclofenac and both agents on proliferation were investigated applying crystal violet staining at a 96 h time point after laminin coating, Figure S7: To measure LDH enzyme activity, the LDH-cytotoxicity assay was performed, Figure S8: Oxygen concentration in the cell cultures was measured using the SDR SensorDish Reader (PreSens Precision Sensing, Regensburg, Germany) for 24 h, Figure S9: (**A**) Extracellular lactate levels after 48 h of treatment with high and low dose metformin. (**B**) pH-levels after metformin and diclofenac treatment (exemplarily shown for U87).
