# Supplementary material for: Combined Modulation of Tumor Metabolism by Metformin and Diclofenac in Glioma
Source: Int J Mol Sci. 2018 Aug 31;19(9):2586. doi: 10.3390/ijms19092586 (PMC6163514; doi:10.3390/ijms19092586)
Supplement: Supplementary file 1 [file ijms-19-02586-s001.pdf]

International Journal of
Molecular Sciences

MDPI

Article

Combined Modulation of Tumor Metabolism by Metformin and Diclofenac in Glioma

Valeria Gerthofer [1], Marina Kreutz [2], Kathrin Renner [2], Birgit Jachnik [1], Katja Dettmer [3], Peter Oefner [3], Markus J. Riemenschneider [4], Martin Proescholdt [5], Arabel Vollmann-Zwerenz [1], Peter Hau [1,†] and Corinna Seliger [1,*,†]

1 Department of Neurology and Wilhelm Sander-NeuroOncology Unit, University Hospital Regensburg, Franz-Josef-Strauss-Allee 11, 93053 Regensburg, Germany; Valeriagerthofer@web.de (V.G); Birgitt.Jachnik@ukr.de (B.J.); Arabel.Vollmann@ukr.de (A.V.-Z.); Peter.Hau@ukr.de (P.H.)
2 Department of Internal Medicine III, University Hospital Regensburg, Franz-Josef-Strauss-Allee 11, 93053 Regensburg, Germany; Marina.Kreutz@ukr.de (M.K.); Kathrin.Renner-Sattler@ukr.de (K.R.)
3 Institute of Functional Genomics, University of Regensburg, Universitätsstraße 31, 93053 Regensburg, Germany; Katja.Dettmer@ukr.de (K.D.); Peter.Oefner@ukr.de (P.O.)
4 Department of Neuropathology, Regensburg University Hospital, Franz-Josef-Strauss-Allee 11, 93053 Regensburg, Germany; Markus.Riemenschneider@ukr.de
5 Department of Neurosurgery, University Hospital Regensburg, Franz-Josef-Strauss-Allee 11, 93053 Regensburg, Germany; Martin.Proescholdt@ukr.de
† Equal contribution
* Correspondence: corinna.seliger@ukr.de; Tel.: 0941-941-88845

Received: 15 July 2018; Accepted: 28 August 2018; Published: date

Abstract: Glioblastoma remains a fatal diagnosis. Previous research has shown that metformin, an inhibitor of complex I of the respiratory chain, may inhibit some brain tumor initiating cells (BTICs), albeit at dosages too high for clinical use.Here, we explored whether a combined treatment of metformin and diclofenac, a non-steroidal anti-inflammatory drug (NSAID) shown to inhibit glycolysis by interfering with lactate efflux, may lead to additive or even synergistic effects on BTICs (BTIC-8, -11, -13 and -18) and tumor cell lines (TCs, U87 and HTZ349). Therefore, we investigated functional effects including proliferation and migration, metabolic effects including oxygen consumption and extracellular lactate levels and effects on the protein level including signaling pathways.Functional investigation revealed synergistic anti-migratory and anti-proliferative effects of the combined treatment with metformin and diclofenac on BTICs and TCs. Signaling pathways did not sufficiently explain synergistic effects. However, we observed that metformin inhibited cellular oxygen consumption and increased extracellular lactate levels, indicating glycolytic rescue mechanisms. Combined treatment inhibited metformin-induced lactate increase.The combination of metformin and diclofenac may represent a promising new strategy in the treatment of glioblastoma. Combined treatment may reduce the effective doses of the single agents and prevent metabolic rescue mechanisms. Further studies are needed to determine possible side effects in humans.

Keywords: glioma; BTICs; metformin; diclofenac; lactate

Supplementary Material:

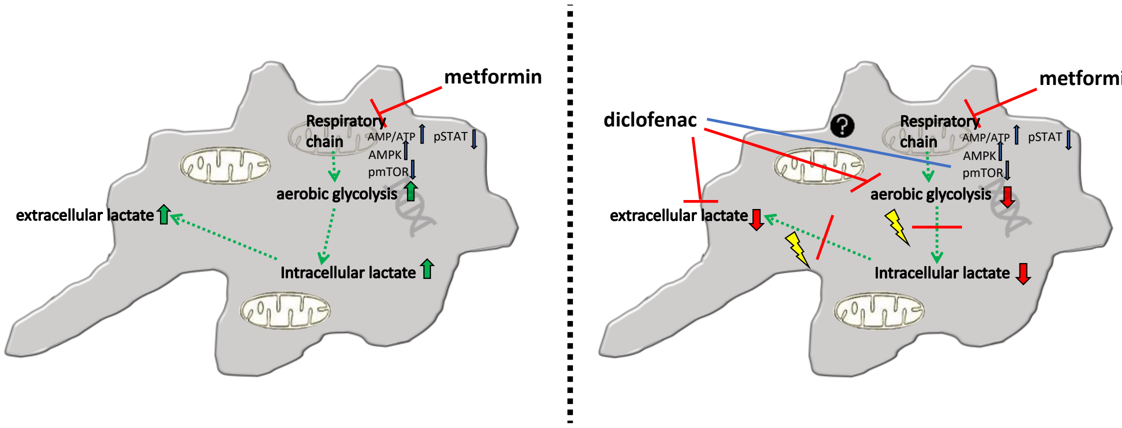

Figure SL1. Graphical abstract summarizing the expected mechanism of action of combined metformin and diclofenac treatment.

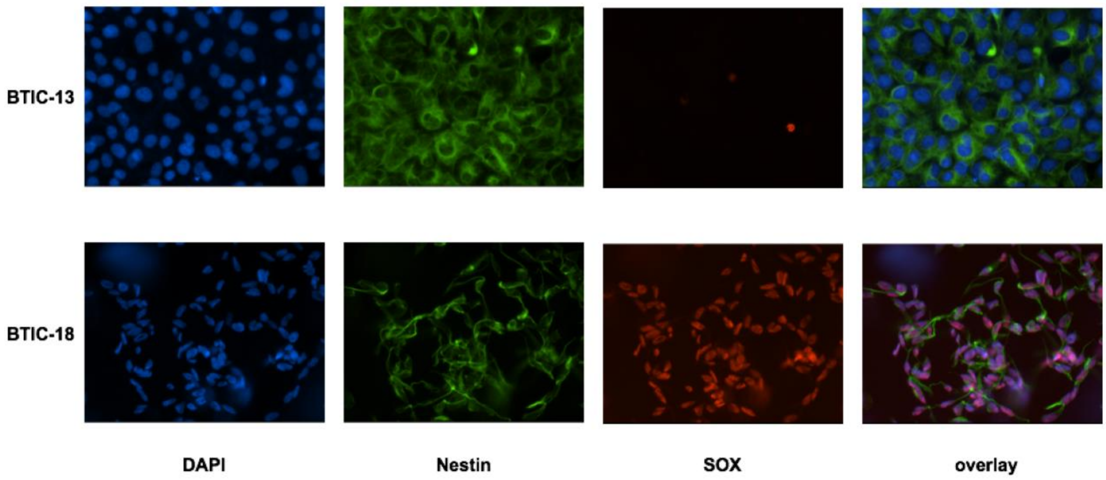

Figure SL2. Immunocytochemical expression of cancer stem cell-markers Nestin and SOX.

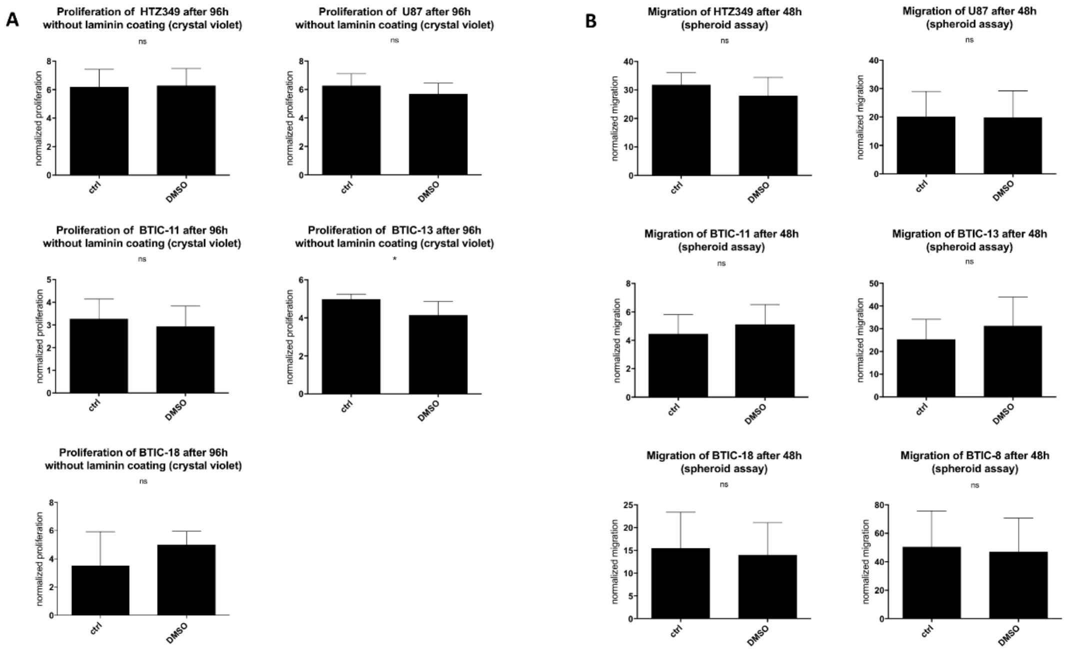

Figure SL3. (**A**)The effects of medium control and DMSO on proliferation were investigated using crystal violet staining at a 96 h time point. (B) Spheroid assays were used to analyze anti-migratory effects at a 48 h time point. Results are expressed as mean ± SD and were analyzed by two-way ANOVA, p = 0.0332 (*), p = 0.0021 (**), p = 0.0002 (***), p = <0.0001 (****) compared pairwise, i.e., the medium vs. DMSO control.

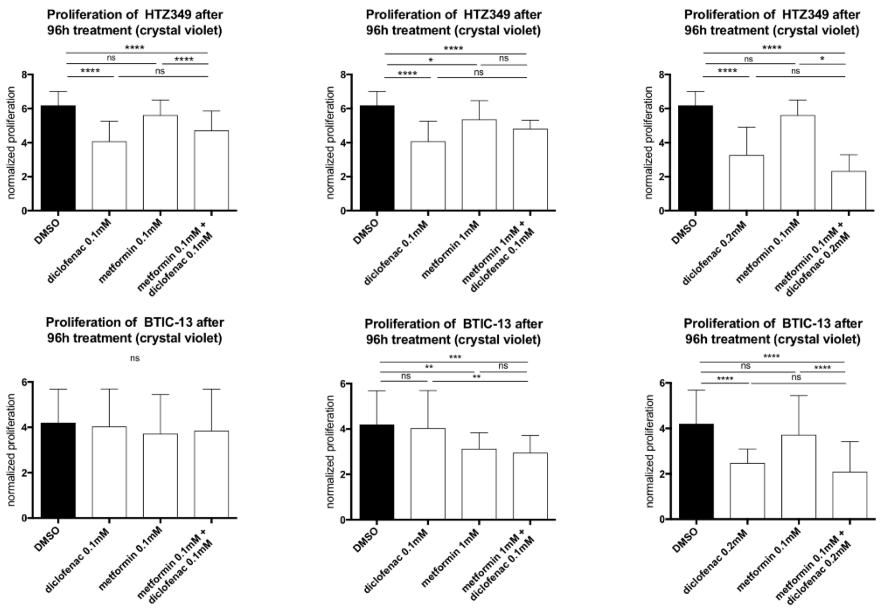

Figure SL4. Cell proliferation of HTZ349 and BTIC-13 after 96 h treatment with different dosages of metformin, diclofenac and both agents was investigated using crystal violet staining. Compared to single treatment with diclofenac no additional inhibition of proliferation was shown. Results are expressed as mean ± SD and were analyzed by two-way ANOVA, * $p < 0.05$, ** $p < 0.01$, *** $p < 0.001$, **** $p < 0.001$ compared pairwise, i.e., the metformin-treated versus metformin and diclofenac condition.

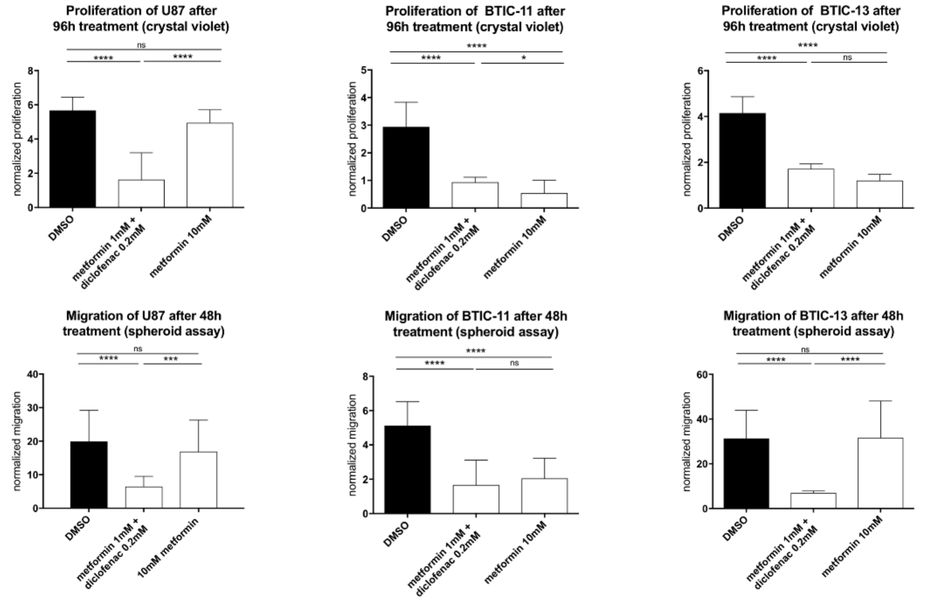

Figure SL5. The effects of metformin, diclofenac and both agents on proliferation were investigated applying crystal violet staining at a 96-h time point. Spheroid assays were used to analyze anti-migratory effects at a 48 h time point.Results are expressed as mean ± SD and were analyzed by two-way ANOVA, $p = 0.0332$ (*), $p = 0.0021$ (**), $p = 0.0002$ (***), $p = <0.0001$ (****) compared pairwise, i.e., the metformin-treated versus metformin and diclofenac condition.

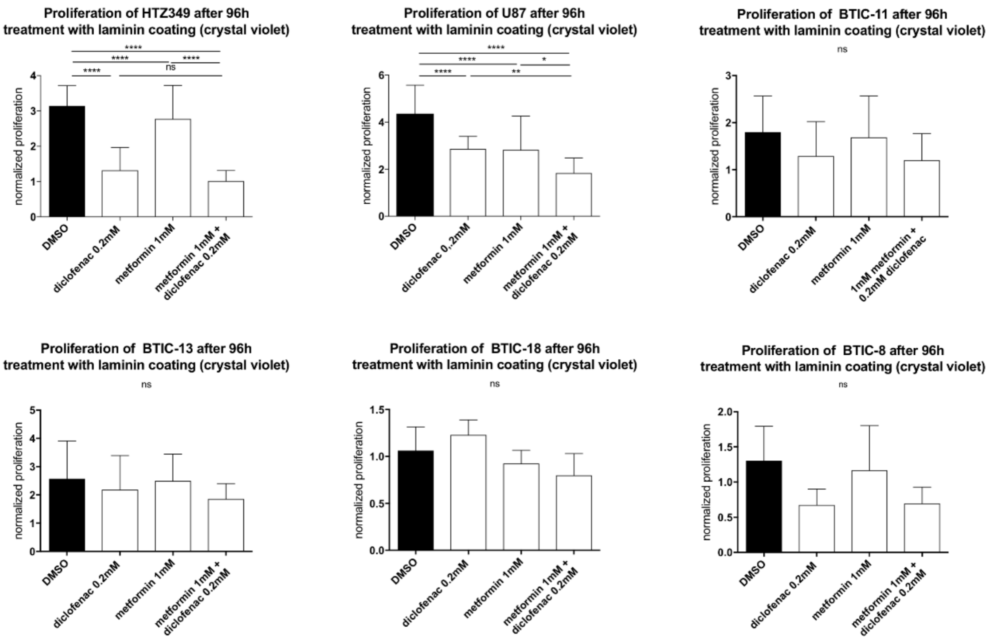

Figure SL6. The effects of metformin, diclofenac and both agents on proliferation were investigated applying crystal violet staining at a 96 h time point after laminin coating. Results are expressed as mean ± SD and were analyzed by two-way ANOVA, $p = 0.0332$ (*), $p = 0.0021$ (**), $p = 0.0002$ (***), $p = < 0.0001$ (****) compared pairwise, i.e., the metformin-treated versus metformin and diclofenac condition.

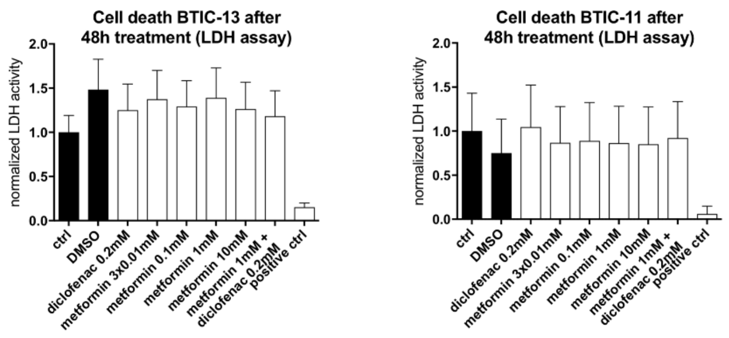

Figure SL7. To measure LDH enzyme activity, the LDH-cytotoxicity assay was performed. Cells were seeded 24 h prior to treatment with 2.5 x 10^3 cells/well in 200 µl serum free media/well and incubated with either the indicated concentrations of metformin, diclofenac or a combination of both, NaOxamat (25 mM) was used as a positive control. 48 h later, LDH activity was measured. Results are expressed as mean ± SD and were analyzed by two-way ANOVA, $p = 0.0332$ (*), $p = 0.0021$ (**), $p = 0.0002$ (***), p = < 0.0001 (****) compared pairwise, i.e., the metformin-treated versus metformin and diclofenac condition.

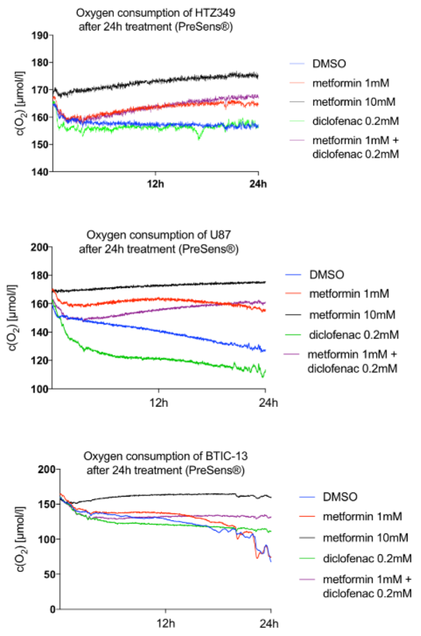

Figure SL8. Oxygen concentration in the cell cultures was measured using the SDR SensorDish Reader (PreSens Precision Sensing, Regensburg, Germany) for 24 h. Cells were seeded considering their stereotypic oxygen consumption in different amounts. Measurements were performed in 60 s intervals.

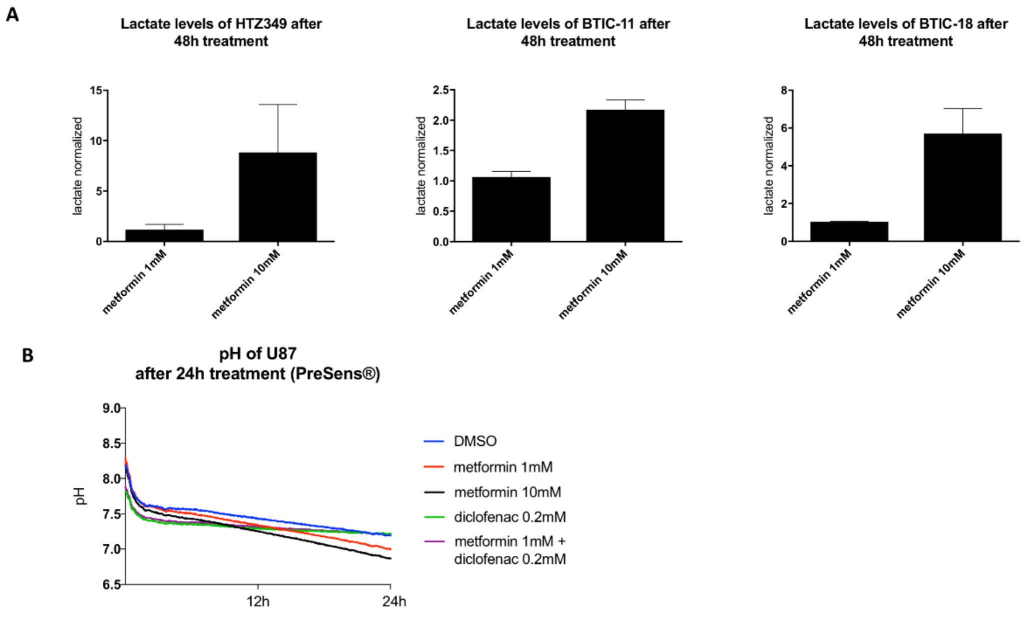

Figure SL9. (**A**) Extracellular lactate levels after 48 h of treatment with high and low dose metformin. Results are expressed as mean ± SD and were analyzed by two-way ANOVA, $p = 0.0332$ (*), $p = 0.0021$ (**), $p = 0.0002$ (***), $p = <0.0001$ (****) compared pairwise, i.e., the metformin-treated versus metformin and diclofenac condition. (**B**) pH-levels after metformin and diclofenac treatment (exemplarily shown for U87).
